# Combining Tumor Treating Fields with Immunotherapy in Pancreatic Ductal Adenocarcinoma: Mechanisms, Preclinical Evidence, and Emerging Therapeutic Synergy

**DOI:** 10.3390/cells15090845

**Published:** 2026-05-05

**Authors:** Douaa Albelal, Hari Krishnareddy Rachamala, Ishita Saha, Santanu Bhattacharya, Debabrata Mukhopadhyay, Hani M. Babiker

**Affiliations:** 1 Division of Hematology and Oncology, Mayo Clinic College of Medicine and Science, 4500 San Pablo Road South, Jacksonville, FL 32224, USA; albelal.douaa@mayo.edu (D.A.); saha.ishita@mayo.edu (I.S.); 2 Department of Biochemistry and Molecular Biology, Mayo Clinic College of Medicine and Science, 4500 San Pablo Road South, Jacksonville, FL 32224, USA; 3 Department of Physiology and Biomedical Engineering, Mayo Clinic College of Medicine and Science, 4500 San Pablo Road South, Jacksonville, FL 32224, USA; 4 Department of Bioengineering, Mayo Clinic College of Medicine and Sciences, 4500 San Pablo Road South, Jacksonville, FL 32224, USA

**Keywords:** tumor treating fields, pancreatic ductal adenocarcinoma, immunotherapy, combined therapy

## Abstract

Tumor Treating Fields (TTFields) represent a novel, non-invasive therapeutic modality in oncology that employs low-intensity, intermediate-frequency alternating electric fields to disrupt mitotic processes and induce cancer cell death. This review integrates mechanistic, preclinical, and emerging clinical evidence supporting the integration of TTFields with immunotherapeutic strategies in pancreatic ductal adenocarcinoma (PDAC). Although immunotherapy has transformed the treatment landscape across multiple malignancies, its efficacy in PDAC remains limited due to the tumor’s dense stroma, immunosuppressive microenvironment, and low immunogenicity. Preclinical investigations suggest that TTFields may potentiate immune-based therapies by enhancing antigen presentation, modulating the tumor microenvironment (TME), and attenuating mechanisms of immune resistance. We highlight studies evaluating TTFields in combination with immune checkpoint inhibitors (ICI), adoptive cellular therapies, and cancer vaccines, emphasizing their potential synergistic effects in PDAC. Clinically, the phase II PANOVA-2 trial demonstrated feasibility and encouraging survival outcomes with TTFields in combination with gemcitabine and nab-paclitaxel, providing the rationale for the ongoing phase III PANOVA-3 trial and the phase II PANOVA-4 trial, which combines TTFields with chemotherapy and atezolizumab. Additional clinical experiences in glioblastoma (GBM) and non-small-cell lung cancer (NSCLC) further substantiate the broader applicability of TTFields as an immunomodulatory adjunct. Remaining challenges include optimizing treatment sequencing, identifying predictive biomarkers, and managing TTFields-associated toxicities. Collectively, current evidence positions TTFields as a promising strategy to augment immunotherapy in PDAC, warranting further translational and clinical investigation to establish its role in reshaping therapeutic paradigms.

## 1. Introduction

PDAC is one of the most lethal human malignancies, characterized by rapid progression, late clinical diagnosis, and profound resistance to conventional therapies [1]. Despite incremental advances in oncology, the prognosis for PDAC remains dismal, with a 5-year overall survival (OS) of only 13.3% [2]. Current standard-of-care regimens, including gemcitabine plus nab-paclitaxel or multi-agent chemotherapy combinations such as FOLFIRINOX and NALIRIFOX, have yielded only modest improvements in progression-free survival (PFS) and OS [3]. However, both intrinsic and acquired resistance to these cytotoxic regimens, combined with significant treatment-related toxicities, severely limit their clinical benefit. Moreover, the unique biology of PDAC, defined by a dense desmoplastic stroma, poor vascularization, and an immunosuppressive TME, acts as a formidable barrier to drug delivery and immune surveillance, further exacerbating therapeutic resistance [4]. These challenges highlight the urgent need for novel therapeutic strategies capable of overcoming resistance mechanisms, modulating the TME, and improving long-term survival outcomes.

TTFields therapy has emerged as an innovative, non-invasive anticancer modality that utilizes low-intensity, intermediate-frequency alternating electric fields to disrupt mitosis in rapidly dividing tumor cells [5]. When applied at frequencies of 100–300 kHz, TTFields interfere with microtubule polymerization and spindle apparatus assembly, both of which are critical for proper chromosome segregation. This interference induces mitotic arrest and catastrophe, ultimately triggering apoptosis or senescence [6,7]. Importantly, TTFields selectively impair proliferating cancer cells while sparing quiescent, non-dividing normal tissue, thereby minimizing systemic toxicity and preserving healthy cell function [8]. Beyond their direct cytotoxic effects, TTFields exert immunomodulatory influences that expand their therapeutic potential. Preclinical studies have shown that TTFields can induce immunogenic cell death (ICD), increase tumor antigen presentation, and enhance leukocyte infiltration within the TME [6]. TTFields have also been reported to increase tumor cell membrane permeability, potentially augmenting the intratumoral penetration of chemotherapeutics and immune-based therapies [9]. This favorable immune remodeling provides a strong rationale for combining TTFields with immune ICI or other immunotherapies. The PANOVA phase II study evaluated the safety and preliminary effectiveness of TTFields combined with gemcitabine alone or gemcitabine plus nab-paclitaxel in patients with advanced pancreatic cancer. TTFields are low-intensity alternating electric fields that disrupt tumor cell division and may enhance chemotherapy response. The study showed that adding TTFields was feasible and generally well tolerated, with manageable toxicity. Early clinical outcomes suggested encouraging survival and disease control compared with historical expectations, supporting further clinical evaluation of TTFields in combination with standard chemotherapy for pancreatic cancer [10]. Encouragingly, clinical studies in GBM and other solid tumors have demonstrated the therapeutic potential of TTFields-based combinations. In PDAC, emerging positive results from advanced-phase clinical studies, including the phase III PANOVA-3 trial, support the ability of TTFields to modulate the TME and enhance immune responsiveness, positioning this approach as a promising component of the evolving immunotherapy landscape [8,11]. The PANOVA-4 (EF-39) pilot study evaluates the feasibility of combining TTFields with atezolizumab, gemcitabine, and nab-paclitaxel as a first-line therapy for metastatic PDAC. Early results indicate that the combination is tolerable and clinically feasible, with manageable safety and preliminary signs of disease control, supporting further investigation in larger clinical trials [12].

## 2. Mechanisms and Action of TTFields in Cancer Therapy

TTFields represent a novel, non-invasive anticancer modality that employs low-intensity (1–3 V/cm), intermediate-frequency (100–300 kHz) alternating electric fields to selectively target dividing cancer cells [5]. The fundamental principle of TTFields lies in their interaction with polarizable macromolecules and organelles essential for mitotic progression [13,14]. During cell division, these alternating fields generate dielectrophoretic forces that disrupt the spatial orientation and function of electrically charged structures, such as microtubules and septins, which are critical for spindle formation and cytokinesis [15]. This interference prevents proper chromosome alignment and segregation, leading to mitotic arrest, chromosomal mis-segregation, and aneuploidy, which ultimately culminate in apoptotic or senescent cell death.

A unique therapeutic advantage of TTFields is their selective activity against rapidly proliferating tumor cells while largely sparing non-dividing somatic tissues, since most normal cells rarely undergo mitosis [8]. Precision in targeting is further enhanced by calibrating field frequency according to tumor cell size and biophysical properties, thereby maximizing therapeutic efficacy while minimizing off-target effects [16]. This selectivity underlies the favorable safety profile of TTFields, allowing long-term, continuous treatment with minimal systemic toxicity. Beyond these direct antimitotic effects, accumulating preclinical evidence suggests that TTFields modulate diverse cellular pathways across different malignancies. In GBM, TTFields impair DNA damage repair pathways, including homologous recombination, thereby sensitizing tumor cells to radiotherapy and genotoxic agents [17]. In NSCLC and mesothelioma, TTFields alter membrane integrity and disrupt key survival signaling cascades, further enhancing cytotoxicity. Importantly, TTFields have also been shown to induce ICD, increase antigen exposure, and reshape the TME, laying the groundwork for synergistic combinations with ICI and other immunotherapies.

**Figure 1:**
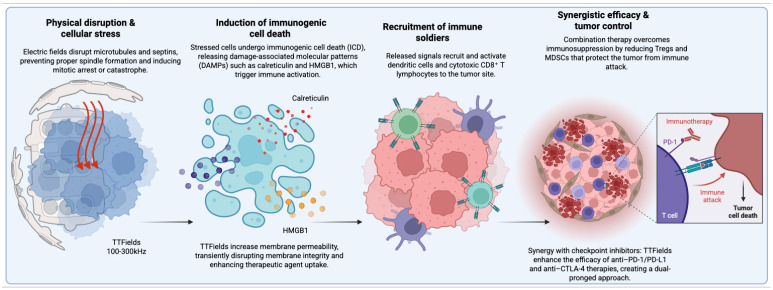
The multifaceted antitumor mechanisms of TTFields and their synergistic interaction with immunotherapy. TTFields (100–300 kHz) induce physical disruption and cellular stress by interfering with microtubules and septins, leading to impaired mitotic spindle formation and mitotic arrest or catastrophe. Concurrently, TTFields enhance membrane permeability, facilitating increased uptake of therapeutic agents. These effects promote ICD, resulting in the release of DAMPs, including calreticulin and HMGB1, which trigger immune activation. The released signals subsequently drive the recruitment and activation of immune cells, including dendritic cells and cytotoxic CD8^+^ T lymphocytes, within the tumor microenvironment. In combination settings, TTFields contribute to overcoming immunosuppression by reducing Tregs and myeloid-derived suppressor cells (MDSCs), thereby enhancing antitumor immunity. Furthermore, TTFields exhibit synergy with immune checkpoint inhibitors (anti–PD-1/PD-L1 and anti–CTLA-4), leading to improved immune-mediated tumor cell killing and overall therapeutic efficacy.

Collectively, these mechanistic insights highlight TTFields as a multifaceted anticancer strategy capable of exerting both direct cytotoxic and immunomodulatory effects (Figure 1). Demonstrated efficacy in GBM, mesothelioma, NSCLC, and more recently PDAC underscores the translational potential of TTFields in highly resistant solid tumors where therapeutic options remain limited [15,17].

## 3. Immunomodulatory Effects of TTFields and Their Therapeutic Role in PDAC

TTFields appear to reprogram the immune microenvironment by suppressing immunosuppressive populations such as regulatory T cells (Tregs) and myeloid-derived suppressor cells (MDSCs), while simultaneously promoting infiltration of cytotoxic CD8^+^ T lymphocytes [10]. This cascade promotes effective priming of adaptive immune responses, transforming TTFields-treated tumors into an “in situ vaccine” that stimulates cytotoxic T lymphocyte activation against malignant cells. The immunologic consequences of TTFields are particularly relevant in PDAC, a malignancy defined by a profoundly immunosuppressive TME. PDAC is characterized by poor effector T-cell infiltration, enrichment of Tregs and MDSCs, and dense stromal barriers that collectively dampen immunotherapy efficacy [18,19]. Preclinical studies in murine models have demonstrated that TTFields can remodel this suppressive niche by enhancing CD8^+^ T-cell infiltration while reducing immunosuppressive Tregs and MDSCs. Such reprogramming of the TME has the potential to mitigate resistance mechanisms that underlie the limited clinical benefit of immune checkpoint blockade therapies in PDAC [20].

In parallel, TTFields have been reported to transiently disrupt tumor cell membrane integrity, thereby improving the intratumoral penetration of therapeutic agents, including monoclonal antibodies, nanoparticle-based drugs, and conventional chemotherapeutics. This property is of particular importance in PDAC, where the desmoplastic stroma and poor vascularization significantly hinder the delivery of both small molecules and immune effector cells [21]. Taken together, TTFields act through dual modalities: (i) direct cytotoxicity via disruption of mitosis and induction of tumor cell death, and (ii) indirect immunologic enhancement through ICD induction, TME modulation, and increased therapeutic penetration. This multifaceted mechanism provides a strong biological rationale for combining TTFields with immunotherapeutic strategies in PDAC. Such integration may enhance antitumor immunity and improve outcomes in a cancer type that has thus far remained refractory to most current immunotherapy approaches [16].

## 4. Preclinical Studies Supporting TTFields’ Immunomodulatory Effects

A substantial body of preclinical research has established that TTFields exert immunomodulatory effects in addition to their direct cytotoxic activity [22]. By inducing ICD, enhancing antigen presentation, and facilitating immune cell infiltration into tumors, TTFields promote a more immunostimulatory TME that potentiates antitumor immunity [6]. These findings provide a mechanistic basis for the rational combination of TTFields with immunotherapeutic strategies in otherwise treatment-resistant malignancies such as PDAC. Voloshin et al. demonstrated that TTFields induce canonical features of ICD in syngeneic murine tumor models, including surface translocation of calreticulin and extracellular release of HMGB1 from dying tumor cells [6]. These damage-associated molecular patterns (DAMPs) promote dendritic cell activation and cross-presentation of tumor antigens, resulting in effective priming of cytotoxic CD8^+^ T lymphocytes and increased intratumoral T-cell infiltration. Functionally, this immune activation was associated with significant tumor growth inhibition, supporting a strong immune-mediated antitumor effect of TTFields [6]. To further interrogate the therapeutic relevance of this immune modulation, Voloshin et al. evaluated TTFields in combination with immune checkpoint blockade using two syngeneic murine models: an orthotopic LLC-1 lung cancer model in C57BL/6 mice and a subcutaneous CT-26 colon carcinoma model in BALB/c mice. Across both models, TTFields combined with anti-PD-1 therapy produced significantly greater tumor control than either monotherapy alone and was accompanied by increased infiltration of leukocytes, including dendritic cells and macrophages; upregulation of PD-L1 on myeloid populations; and elevated interferon-γ production by cytotoxic CD8^+^ T cells. Mechanistic insights in this study were derived from analyses of DAMP release, autophagy induction, dendritic cell maturation, and immune phenotyping, rather than transcriptomic approaches.

The immunologic activity of TTFields has been further substantiated in preclinical models of NSCLC. When combined with ICI, TTFields exert additive antitumor effects both in vitro and in vivo without compromising T-cell-mediated cytotoxicity. Beyond anti-PD-1, TTFields also potentiated broader immune checkpoint blockade (ICB), including anti-PD-L1 and anti-CTLA-4, resulting in greater tumor suppression, increased infiltration of cytotoxic CD8^+^ T cells, and elevated interferon-γ production. Together, these findings position TTFields as a robust immune-sensitizing modality capable of augmenting checkpoint inhibition in NSCLC. Murine models of lung cancer and melanoma demonstrated that TTFields combined with dual ICB (anti-PD-1 plus anti-CTLA-4) achieved superior tumor regression and extended survival relative to monotherapy arms. Mechanistically, this combinatorial efficacy was linked to enhanced dendritic cell maturation, amplified cytotoxic T-cell activation, and depletion of immunosuppressive populations such as Tregs and MDSCs. Together, these studies highlight TTFields as an immune-priming modality capable of converting “cold” tumors into immune-permissive states. TTFields directly affect tumor cell biophysics in ways that further support combination immunotherapy. TTFields transiently disrupt tumor cell membrane integrity, thereby improving the uptake of macromolecular therapeutics such as nanoparticles and monoclonal antibodies and facilitating deeper distribution of checkpoint inhibitors or antibody-based drugs. TTFields may help overcome both the physical and immunologic barriers that underpin resistance to immunotherapy.

Collectively, these preclinical studies underscore the multifaceted immunomodulatory role of TTFields. By inducing ICD, reshaping the immune composition of the TME, and enhancing the delivery of immune-targeting agents, TTFields establish a strong rationale for their integration with ICB and other immunotherapeutic modalities. Such combinations hold particular promise in PDAC, where profound immunosuppression and stromal barriers have historically blunted the efficacy of immune-based therapies.

## 5. Preclinical and Clinical Evidence Supporting TTFields Immunotherapy Synergy in PDAC

An expanding body of preclinical and clinical evidence supports the hypothesis that TTFields enhance immunotherapy efficacy through complementary and synergistic mechanisms. Beyond their established antimitotic activity, TTFields have emerged as potent modulators of both innate and adaptive antitumor immunity, reshaping the tumor–immune interface and sensitizing tumors to immune checkpoint inhibition [23]. Mechanistically, TTFields induce immunogenic stress responses that promote ICD. Chen et al. demonstrated that TTFields disrupt the nuclear envelope, leading to cytosolic micronucleus formation and activation of the cGAS–STING pathway and AIM2 inflammasome, resulting in type I interferon production and proinflammatory signaling. This dual activation drives durable antitumor immunity and immunological memory, achieving long-term tumor control in preclinical GBM models [24]. Supporting these findings, Low et al. showed that TTFields induce DNA damage and micronucleus formation, reinforcing cGAS–STING-mediated immune activation and suggesting an in situ vaccination effect in patients [25].

Further studies have confirmed that TTFields promote ICD characterized by calreticulin exposure and HMGB1 release, enhancing dendritic cell activation, antigen presentation, and CD8^+^ T-cell infiltration [6]. In NSCLC and other solid tumor models, TTFields also upregulate IFNγ/STAT1/IRF1 signaling and chemokines such as CCL2, CCL8, CXCL9, and CXCL10, thereby facilitating recruitment of effector T cells and remodeling the TME [23]. When combined with ICB, including anti-PD-1 and anti-CTLA-4 therapies, TTFields significantly enhance tumor regression, prolong survival, and reduce immunosuppressive populations such as regulatory T cells and myeloid-derived suppressor cells [26]. Importantly, TTFields also exert physical effects that complement their immunologic activity. They transiently disrupt tumor cell membrane integrity, improving intratumoral delivery of therapeutic agents such as monoclonal antibodies, nanoparticles, and small molecules, an advantage particularly relevant in desmoplastic tumors like PDAC, where stromal barriers limit drug penetration [15,27]. Notably, TTFields maintain T-cell effector functions, including interferon-γ production and cytotoxicity, supporting their compatibility with T cell-based therapies such as CAR-T cells [28].

Clinically, TTFields have already demonstrated efficacy in GBM and malignant pleural mesothelioma, improving progression-free and overall survival without significant systemic toxicity [29]. In metastatic non-small-cell lung cancer, the phase III LUNAR trial showed that combining TTFields with standard systemic therapy—including ICI and chemotherapy—significantly improved overall survival compared with standard therapy alone, providing robust clinical validation for TTFields-based combination strategies [30]. Although direct clinical evidence in PDAC remains limited, early translational and clinical observations suggest that TTFields combined with immunotherapy or chemotherapy may yield additive or synergistic benefits. These combinations have been associated with increased CD8^+^ T-cell infiltration, improved tumor control, and enhanced disease stabilization in immunologically “cold” tumors [31,32].

Collectively, these findings establish TTFields as multifunctional immunogenic modulators capable of inducing ICD, activating innate immune pathways, enhancing adaptive immune responses, and improving therapeutic delivery. By “heating up” the immunosuppressive PDAC microenvironment, TTFields provide a strong biological rationale for combination with immunotherapies. This strategy is now advancing from preclinical validation to clinical investigation and holds promise for overcoming immune resistance in PDAC and other refractory malignancies. Key preclinical studies evaluating TTFields immunotherapy synergy are summarized in Table 1.

## 6. Clinical Trials of TTFields in Combination with Immunotherapy

The clinical development of TTFields has advanced rapidly from GBM into other solid tumors, with an increasing focus on combinatorial strategies with ICIs. These ongoing and completed studies are designed to assess safety, efficacy, and immunologic mechanisms, while also exploring biomarker-driven predictors of response. Together, they provide a growing foundation for the integration of TTFields into the evolving immuno-oncology landscape. LUNAR-2 (NCT06216301) is a Phase III trial evaluating TTFields in combination with pembrolizumab and platinum-based chemotherapy in metastatic NSCLC, incorporating immune-correlative analyses to characterize treatment-associated immunologic changes. In parallel, PANOVA-4 (NCT06390059) is a Phase I/II study investigating TTFields combined with atezolizumab, gemcitabine, and nab-paclitaxel in metastatic PDAC, with a focus on safety, immune modulation, and early efficacy signals. Together, these studies represent the first TTFields–ICI clinical trials in thoracic and gastrointestinal malignancies with formal immune-profiling endpoints. The pivotal Phase III LUNAR trial tested TTFields combined with standard systemic therapy (ICI or docetaxel) in metastatic NSCLC after platinum-based chemotherapy. TTFields significantly improved median OS (13.2 vs. 9.9 months), with the most pronounced benefit observed in patients receiving ICIs (18.5 vs. 10.8 months). These findings culminated in the first FDA approval of TTFields for metastatic NSCLC in 2024, marking a milestone for TTFields beyond GBM.

The success of TTFields in GBM continues to inform broader oncologic applications. The EF-14 trial established survival benefits of TTFields with adjuvant temozolomide, leading to global consensus guidelines. The Canadian Delphi panel endorsed TTFields with temozolomide for both newly diagnosed and recurrent GBM, aligning with National Comprehensive Cancer Network (NCCN) recommendations [33]. Ma et al. further mapped randomized controlled trials in elderly GBM, demonstrating the feasibility and clinical benefit of TTFields in this subgroup while underscoring the absence of immunotherapy-based RCTs, thereby reinforcing the rationale for TTFields–immunotherapy studies [34]. He et al. performed a comprehensive analysis of glioma clinical trials registered between 2005 and 2021, revealing a decline in chemotherapy- and radiotherapy-focused studies and a corresponding rise in immunotherapy-oriented and TTFields-based trials. This shift reflects the global recognition of TTFields as an emerging standard modality [35].

Emerging evidence suggests broader applicability of TTFields in solid tumors. Yue et al. provided the first preclinical evidence for TTFields in biliary tract cancer (BTC), showing suppression of proliferation and induction of ICD biomarkers (calreticulin exposure, HMGB1 release, extracellular ATP secretion). These findings have prompted initiation of a Phase Ib trial (NCT06611345) combining TTFields with immunotherapy in BTC [36]. Case reports further highlight TTFields’ potential in refractory malignancies. Torres Velasco et al. described a hepatocellular carcinoma (HCC) patient refractory to nivolumab who achieved 39 months of disease stabilization when treated with TTFields plus sorafenib. This exceptional response suggests that TTFields may prolong therapeutic durability, even in immunotherapy-experienced settings [37]. Notably, the NRG-BN007 trial, which investigated dual checkpoint blockade with nivolumab and ipilimumab in newly diagnosed MGMT-unmethylated GBM, excluded TTFields from the immunotherapy arm due to concerns about overlapping skin toxicities [38]. This highlights the need for thoughtful integration strategies to optimize tolerability in TTFields–ICI combinations. Su et al. conducted a network meta-analysis of bevacizumab-based regimens in recurrent GBM, where TTFields plus bevacizumab ranked favorably for 6-month OS [39]. Such evidence suggests that TTFields are already being incorporated into multimodal therapeutic frameworks and reinforces their relevance in recurrent disease settings.

Collectively, these trials and analyses establish TTFields as a clinically validated, immunomodulatory modality with expanding applications beyond GBM. Ongoing studies in PDAC, NSCLC, and gastrointestinal cancers will be critical to defining the therapeutic role of TTFields in combination with ICIs. While practical barriers such as toxicity management and cost-effectiveness remain, the convergence of clinical evidence strongly supports TTFields as a next-generation platform therapy capable of synergizing with immunotherapy across multiple solid tumors. Clinical trial characteristics are summarized in Table 2.

These ongoing studies are crucial for translating promising preclinical findings into potential new treatment paradigms for difficult-to-treat cancers. Emerging Evidence and Translational Insights: The immunologic potential of TTFields has garnered increasing attention within the oncology community, with accumulating preclinical and early clinical evidence supporting immunomodulatory activity, and with studies synthesizing preclinical and early clinical evidence. Collectively, these analyses underscore TTFields as more than a cytostatic modality, positioning them as active immunomodulators capable of reshaping the tumor–immune interface in solid malignancies [40]. Preclinical investigations have demonstrated that TTFields can induce ICD, enhance tumor antigen availability, and promote dendritic cell maturation. These effects are accompanied by activation of pro-inflammatory signaling cascades, including the release of DAMPs such as calreticulin, ATP, and HMGB1. These molecular events contribute to the recruitment and activation of cytotoxic lymphocytes, thereby potentiating antitumor immunity. Importantly, these properties could help counteract the profoundly immunosuppressive TME that characterizes refractory malignancies such as PDAC. Building on these mechanistic insights, a strong biological rationale has emerged for combining TTFields with ICB in solid tumors historically resistant to immunotherapy [10]. TTFields appear to possess a dual mechanism of action: direct tumor cytotoxicity via mitotic disruption, and indirect immune potentiation through TME reprogramming. By disrupting tumor cell division while simultaneously amplifying immune responsiveness, TTFields may help overcome the limited efficacy of immunotherapies in “cold” tumors. Notably, early preclinical studies have demonstrated synergy (Figure 1) between TTFields and ICIs, together with emerging safety data, as justification for advancing these combinations to clinical testing across multiple tumor types, including PDAC.

Although large-scale randomized clinical trials of TTFields combined with immunotherapy in PDAC are not yet available, the convergence of preclinical studies and mechanistic plausibility has generated significant translational momentum. The steady rise in ongoing clinical trials exploring TTFields–ICI combinations across gastrointestinal, thoracic, and central nervous system malignancies further reflects the growing recognition of TTFields as a platform therapy with broad immunotherapeutic potential. Taken together, these mechanistic and translational findings provide a strong scientific and clinical rationale for continued investigation of TTFields as an immunomodulatory adjunct. In PDAC and other immune-refractory cancers, TTFields may offer a transformative strategy to convert immunologically “cold” tumors into immune-permissive states, thereby enhancing the efficacy of checkpoint blockade and other next-generation immunotherapies.

## 7. Challenges and Future Directions for TTFields–Immunotherapy Combinations in PDAC

Despite encouraging preclinical evidence and the initiation of early-phase clinical trials, several critical challenges must be addressed before the therapeutic potential of TTFields in combination with immunotherapy can be fully realized in PDAC, particularly in the context of rapidly evolving systemic therapies. Although TTFields have demonstrated immunomodulatory activity in multiple preclinical tumor models, clinical validation in PDAC remains at a nascent stage. Most current trials are early-phase and enrolling PDAC patients within broader gastrointestinal cancer cohorts, limiting the ability to derive tumor-specific conclusions. To date, no completed randomized controlled trials (RCTs) have established the clinical efficacy of TTFields immunotherapy combinations in PDAC. This highlights the need for carefully designed, biomarker-enriched clinical studies incorporating integrated immune monitoring and survival endpoints, although the feasibility of large PDAC-specific RCTs may be increasingly challenged by the rapid emergence of effective molecularly targeted therapies [11] The optimal temporal integration of TTFields with ICI is not yet defined. Preclinical models suggest that concurrent administration enhances synergistic immune activation, but clinical studies have not systematically evaluated whether initiating TTFields prior to, during, or after immunotherapy maximizes therapeutic benefit. Moreover, the impact of variations in field intensity, application duration, and scheduling immune priming remains unexplored. Carefully designed clinical trials are required to establish evidence-based sequencing strategies that optimize both immunologic and clinical outcomes [41]. A major obstacle to personalized TTFields–ICI therapy is the absence of validated predictive biomarkers. While candidate markers such as MHC class I expression, interferon-γ signatures, and indicators of immunogenic cell death have been proposed, none have been prospectively validated in PDAC. Considering the profound heterogeneity of PDAC in terms of tumor immunogenicity and stromal composition, biomarker-driven patient selection will be essential for trial design, treatment stratification, and clinical decision-making [42]. PDAC is characterized by a profoundly immunosuppressive and desmoplastic TME, marked by dense fibrosis, infiltration of myeloid-derived suppressor cells, and polarization of tumor-associated macrophages toward an M2 phenotype. While TTFields have been shown to enhance antigen presentation and promote T-cell infiltration, it remains uncertain whether these effects alone are sufficient to overcome the entrenched immune exclusion of PDAC. Mechanistic studies utilizing patient-derived organoids, co-culture systems, and spatial immune profiling will be essential to delineate the extent of TME remodeling induced by TTFields alone and in combination with emerging targeted therapies [43]. Recent progress in direct RAS inhibition is beginning to change how KRAS-mutant PDAC is approached therapeutically. Daraxonrasib (RMC-6236), a selective RAS(ON) multi-target inhibitor, has shown promising activity in early clinical studies involving heavily pretreated patients with metastatic PDAC harboring KRAS G12X mutations. These phase 1 data demonstrated objective response rates in the 30–36% range, median PFS of approximately 8–9 months, and a generally manageable safety profile. Together, these results supported FDA Breakthrough Therapy Designation and the initiation of ongoing phase 3 trials evaluating daraxonrasib both as monotherapy and in combination with chemotherapy [44].

Importantly, the impact of RAS inhibition may extend beyond direct tumor growth suppression. Preclinical studies of KRAS-targeted agents, including KRAS^G12D inhibitors, suggest that blocking oncogenic RAS signaling can reshape the tumor–immune microenvironment. These effects include increased infiltration of CD8^+^ T cells, reduction in immunosuppressive myeloid populations, and enhanced sensitivity of tumor cells to T-cell-mediated killing, driven in part by changes in inflammatory and interferon-related pathways. By reversing key mechanisms of RAS-driven immune exclusion, RAS inhibition may help create a tumor milieu that is more permissive to immune engagement [45].

Against this backdrop, there is a strong biological rationale for exploring combination strategies that integrate RAS inhibitors with TTFields and ICB. RAS inhibition may reduce intrinsic tumor resistance and stromal-mediated immune suppression, TTFields may promote ICD and tumor antigen release, and ICB may then amplify effective antitumor T-cell responses. While the feasibility of large, randomized trials in PDAC may be increasingly constrained by the rapidly evolving treatment landscape, well-designed translational studies incorporating immune profiling and molecular stratification will be critical to test these hypotheses and identify the patients most likely to benefit from such multimodal approaches.

However, TTFields therapy requires continuous administration through wearable transducer arrays for 12–18 h per day to achieve therapeutic efficacy. Although generally well tolerated, adherence may become challenging when combined with chemotherapy or immunotherapy regimens. Furthermore, the substantial cost of TTFields devices raises questions about cost-effectiveness, particularly in a malignancy such as PDAC, where overall therapeutic responsiveness has historically been limited [46]. To translate the mechanistic promise of TTFields immunotherapy synergy into meaningful clinical benefit for PDAC, several avenues of investigation must be prioritized. Conducting biomarker-driven and molecularly stratified clinical studies that integrate immune monitoring and survival endpoints will be essential to establish efficacy beyond early-phase investigations. Parallel efforts should focus on the development and validation of predictive biomarkers capable of guiding patient selection and optimizing treatment stratification, thereby addressing the marked heterogeneity of PDAC. Furthermore, systematic evaluation of treatment sequencing, field intensity, and application duration in relation to immunotherapy is required to define the optimal therapeutic window and maximize immune priming. It will also be critical to explore rational combination strategies that integrate TTFields not only with immunotherapy but also with chemotherapy, stromal modulation, and molecularly targeted therapies, as these multimodal regimens may overcome the profound immunosuppressive microenvironment of PDAC. Finally, real-world implementation studies assessing feasibility, patient adherence, and cost-effectiveness will help determine the translational potential of TTFields in clinical oncology. As these research directions advance, TTFields may evolve from a predominantly cytostatic modality into a powerful tool for immune reprogramming, offering new therapeutic opportunities in one of the most treatment-resistant malignancies.

## 8. Conclusions

PDAC remains a formidable therapeutic challenge due to its intrinsic resistance to systemic therapies and profoundly immunosuppressive microenvironment. TTFields, with their dual cytostatic and immunomodulatory effects, present a novel opportunity to reshape the tumor–immune interface and sensitize PDAC to immunotherapy. Preclinical studies offer compelling evidence that TTFields enhance ICD, promote immune cell infiltration, and synergize with ICB in solid tumors. Although clinical data are still preliminary, ongoing trials highlight the translational potential of TTFields in combination with immunotherapy. Future research must prioritize validating efficacy in PDAC-specific patient cohorts, identifying predictive biomarkers, and optimizing treatment regimens. Addressing these challenges may enable TTFields immunotherapy combinations to redefine the therapeutic landscape of PDAC, providing renewed hope for one of oncology’s most treatment-refractory malignancies.

## Figures and Tables

**Table 1:** Summary of key preclinical studies on TTFields and immunotherapy synergy.

Study	Experimental Model	Key Findings	Evidence of Synergistic Effect with Immunotherapy
Voloshin et al. (2020) [6]	Murine glioma	Induction of immunogenic cell death (ICD), increased CD8^+^ T-cell infiltration, dendritic cell activation	Yes
Chen et al. (2022) [24]	Murine lung cancer and melanoma	Prolonged survival, increased CD8^+^ T cells, decreased Tregs and MDSCs when combining TTFields + ICI	Yes
Gera et al. (2015) [15]	In vitro human cancer cells	Enhanced tumor cell membrane permeability, increased uptake of antibodies and nanoparticles	Indirect support facilitates drug delivery

**Table 2:** The summary below outlines key design features of these trials.

Trial ID	Phase	Cancer Type	Combination	Status	Focus Areas
NCT06216301	III	NSCLC	TTFields + pembrolizumab + platinum chemotherapy	Recruiting	Immune profiling; treatment-associated immunologic changes
NCT06390059	I/II	PDAC	TTFields + atezolizumab + gemcitabine + nab-paclitaxel	Active (not recruiting)	Safety; immune modulation; early efficacy signals

## Data Availability

Availability Statements are available in section “MDPI Research Data Policies” at https://www.mdpi.com/ethics, accessed on 27 April 2026.
